# The change in capacity and service delivery at public and private hospitals in Turkey: A closer look at regional differences

**DOI:** 10.1186/1472-6963-10-300

**Published:** 2010-11-01

**Authors:** Hediye AD Aksan, Işıl Ergin, Zeliha Ocek

**Affiliations:** 1 Ege University Medical Faculty Department of Public Health, 35100 Bornova, Izmir Turkey

## Abstract

**Background:**

Substantial regional health inequalities have been shown to exist in Turkey for major health indicators. Turkish data on hospitals deserves a closer examination with a special emphasis on the regional differences in the context of the rapid privatization of the secondary or tertiary level health services.

This study aims to evaluate the change in capacity and service delivery at public and private hospitals in Turkey between 2001-2006 and to determine the regional differences.

**Methods:**

Data for this retrospective study was provided from Statistical Almanacs of Inpatient Services (2001-2006). Hospitals in each of the 81 provinces were grouped into two categories: public and private. Provinces were grouped into six regions according to a development index composed by the State Planning Organisation. The number of facilities, hospital beds, outpatient admissions, inpatient admissions (per 100 000), number of deliveries and surgical operations (per 10 000) were calculated for public and private hospitals in each province and region. Regional comparisons were based on calculation of ratios for Region 1(R1) to Region 6(R6).

**Results:**

Public facilities had a fundamental role in service delivery. However, private sector grew rapidly in Turkey between 2001-2006 in capacity and service delivery. In public sector, there were 2.3 fold increase in the number of beds in R1 to R6 in 2001. This ratio was 69.9 fold for private sector. The substantial regional inequalities in public and private sector decreased for the private sector enormously while a little decrease was observed for the public sector. In 2001 in R1, big surgical operations were performed six times more than R6 at the public sector whereas the difference was 117.7 fold for the same operations in the same regions for the private sector. These ratios decreased to 3.6 for the public sector and 13.9 for the private sector in 2006.

**Conclusions:**

The private health sector has grown enormously between 2001-2006 in Turkey including the less developed regions of the country. Given the fact that majority of people living in these underdeveloped regions are uninsured, the expansion of the private sector may not contribute in reducing the inequalities in access to health care. In fact, it may widen the existing gap for access to health between high and low income earners in these underdeveloped regions.

## Background

During the last three decades in many countries, the scope of the private practice in health enhanced and market elements were introduced into health care financing. The governments started programs that extended cost sharing, introduced performance-related payment schemes, reconstructed the role of state and private agents in health care and many other programs [1]. Turkey has been one of these countries and health reforms have been in the agenda since 1980.

Turkey's economy is among the world's twenty largest, with a GDP per capita exceeding US$8,000. However, health status in Turkey has not shown satisfactory development compared to its counterparts in OECD [2-4]. The life expectancy in Turkey is slightly below the average for its income level, when it is compared to other upper-middle income countries [2-5]. Health indicators such as infant mortality and mother mortality are at very high levels compared to that of the countries in its region and shows significant regional and urban-rural health inequalities [3]. In 2003 Demographic Health Survey (DHS), it was reported that Infant Mortality Rate(IMR) was 22 per 1,000 live births in the west compared to 41 per 1,000 in the eastern part of the country[6].

### Social security and hospital services in Turkey before Health Transformation Programme(HTP)

Until 1945 the health system was mainly financed through Ministry of Finance and MoH and local governments. Between 1945-1971 new organizations were established for health financement. The social security system was at the core of public health financing arrangements until HTP. In the system before HTP, there were three separate health insurance funds: *i) *SSK for blue and white-collar workers in the public and private sectors; *ii) Bag -Kur *(the Social Security Organisation for Artisans and the Self-Employed); and *iii) Emekli-Sandigi(*Government Employees Retirement Fund -GERF). Active civil servants were not included in GERF and their expenses were directly financed from the state budget. After 1992 Green Card was introduced to the system which provided health benefits to the poor and uninsured. According to Turkey Household Budget Survey which is conducted by the Turkish Statistical Institute (TUIK), the percentage of the population covered by any of the above health insurances in 2003 was 64% (3). Contrary to the hospital services, no payment was required from citizens for primary care, even if not covered under social security.(3).

Although Turkey has always had a mixed health service delivery system consisting of public and private providers, the vast majority of the health service provision was through the public sector. Until 1990 the three key public service providers were the Ministry of Health, SSK and the universities through university hospitals. The public hospitals were mainly financed from social security institutions, general budget through Ministry of Finance and MoH and local governments [7].

Before the late 1980s, a few private hospitals, mainly in Istanbul, were established. However during the economic liberalization of the late 1980s, the encouragement of the private sector investment in the health services was an important item which was successfully implemented with the help of generous government subsidies (ie. incentive premiums for those who were to build private hospitals). This resulted in the building of many private hospitals by the end of 1990s. Many of these new hospitals offered integrated diagnostic and outpatient services and luxurious inpatient hotel facilities to attract selfpaying, fee-for-service patients [3,7,8]. The government agencies started to purchase some of their services from private hospitals by the end of 1990's. For example, SSK purchased cardiovascular surgical services from private hospitals. Up until that stage, the private hospital revenues were mainly from out of pocket payments, private insurance and with a small share from government contracts. But after HTP, the share of government spending through these contracts, became a substantial source of revenue for private hospitals [3].

### Hospital Services and Health Transformation Programme (HTP) between 2003-2006

The HTP was conceived as a ten-year reform programme covering the period 2003-13 [3]. The program was declared to address the long-standing problems in the Turkish health system: lagging health outcomes as compared to other OECD and middle-income countries; inequities in access to health care; fragmentation in financing and delivery of health services, which contributes to inefficiency and undermines financial sustainability; and poor quality of care and limited patient responsiveness [8,9].

Major steps taken with the onset of Health Transformation Programme (HTP) affecting hospital services were:

#### Changes in finance of health

General Health Insurance system(GHI) was established in 2008 (but has been at the centre of the policies since 2004) to combine different social security schemes under one umbrella. The revolving funds financed from social security insurances became the main financial resource of hospitals[3,9].

#### Changes in provision

SSK hospitals were transferred to MoH. The extend of the outpatient and inpatient services in which the enrolees of public social security institutions were allowed to access from private health facilities were enhanced [8,3].

At 2005, the provision of SSK members to access private facilities for outpatient and inpatient health services were enhanced, while this was still not allowed for the rest of the security schemes. The scope of these health services and co-payments differed within facilities according to their contracts. In 2007 other public social security institutions started to get into contracts with private health facilities.

#### Changes healthcare management and operations

MoH was restructured with the objective of strengthening its stewardship function. Individual performance based payment systems were implemented in MoH hospitals. Some health services (food preperation, laundry and cleaning, some laboratory and some radiodiagnostic services) of hospitals were outsourced [3,8].

#### Changes in healthcare investment

Government incentives for private hospital investment were increased [3,8].

### Inequalities

Substantial regional health inequalities have been shown to exist in Turkey across major health indicators and in provision of health services. The populations in the east, middle and southeast regions compared with other regions of the country show substantially lower education levels, lower income patterns, and higher unemployment levels. Deman-led forces are weak. The utilization of health services has been much lower in these low developed regions regardless of the health indicators which are markedly worse than the country average. The private investors have not been too eager to invest in these areas of low socioeconomic development, until the last decade during which the government policies and incentives had reversed this situation. These regions have enjoyed a significant increase in private health investment. However self payments for uninsured or co-payments for the insured for utilizing private care do not ease access to health care especially for the poor, which forms the majority of the population in these regions[3,8,9].

To achieve equity in access to health care has been claimed to be one of the four major aims of HTP. Thus, the characteristic of the distribution of capacity and service delivery to reach everyone is an important component of this issue.

Turkish data on hospitals deserves a closer examination with a special emphasis on the regional differences in the context of the rapid privatization of the secondary or tertiary level health services.

The data about the nature of the change in this distribution for each region considering public and private share is scarce. However the official data from Statistical Almanacs of Inpatient Services is representative and most reliable national data of public and private hospital services. An accurate characterization of the change throughout the years for each region by closely examining this data may enable policy makers and health planners to mobilize and allocate resources for appropriate choices if achieving equity is the main goal.

This study aims to present i)the characteristics of health service capacity and service delivery for public and private in the country ii) the change in capacity and service delivery for each region between 2001 to 2006 for public and private and iii) the regional differences during this period.

## Methods

### Variables and data sources

In this retrospective study, data provided in the Statistical Almanacs of Inpatient Services (2001-2006) published by the MoH was used[10-15]. This is the only available national data set for hospital services in private and public sectors in the country. The dataset included all the hospitals in Turkey between 2001-2006. In these Almanacs each year's data is presented separately for each province. The hospitals in each of the 81 provinces have been grouped into public and private.

The public hospitals included are the hospitals run by Ministry of Health, Social Security Institution (before 2005), public universities, Ministry of Defence, Municipalities, and other public institutions. The hospitals run by the Social Security Institution were merged with the MoH hospitals in 2005. Private hospitals included are those owned by private Turkish citizens and established as a corporation, hospitals owned by minorities and private university hospitals.

**Figure 1:**
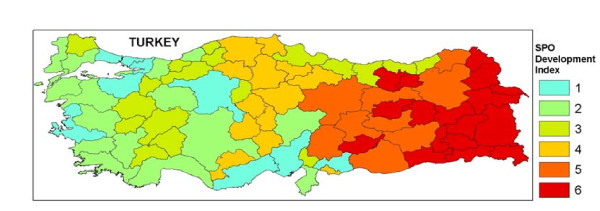
**Provinces of Turkey according to SPO Development Index**.

The cities were grouped into six regions according to an index developed by State Planning Organisation (SPO) (Figure 1) [16]. The number of facilities and hospital beds per 100 000, the number of outpatient admissions, inpatient admissions per 100 000, the number of deliveries and surgical operations per 10 000 have been calculated for each province and region.

The variables used for comparison were calculated for the two categories "public" and "private" for each province. For every variable, the annual change and the six-year change in percentages have been separately evaluated for public and private sector.

The index of SPO was developed for MoH by taking into account the variables such as sociodemographic, financial, educational characteristics, and status of agriculture, industry, employment and health [16,17]. The regions are listed in the order of development, the most developed region being Region 1 and the least developed region being Region 6.

For example, for Izmir province which is in Region 1, total fertility rate was 1,75, number of physicians were 22 per 10000, illiteracy rate was 8,1% and GDP per capita was 2 696 YTL. However for Hakkari province which is in Region 6, total fertility rate was 6,69, number of physicians were 3,55 per 10000, illiteracy rate was 29,3% and GDP per capita was 696 YTL[17].

### Measuring inequalities

In this study, relative differences have been expressed as Rate Ratios. That is, as a proportion of the value obtained for a reference category (Rate ratio of lowest versus highest group). Higher rate ratios show higher inequalities. The reference category has been the most developed region: Region 1. Each rate is the frequency with which numbers of cases (beds, surgeries, deliveries etc) are present (numerator) for a certain number of people (denominator).

### The categorization of MoH for major, intermediate and small operations is as follows

**Major operations **were cardiac transplantation, kidney transplantation, small intestine transplantation, hematopoietic cell transfer, Aorta replacement mitral valve reconstruction, hepatectomy, pharyngolaryngectomy, mediasten cyst/tumor excision, by-pass graft, excision of malign skin tumors, open rhynoplasty, laryngectomy, mandibular or maxillar reconstruction, bone graft etc.

**Intermediate operations **were muscular flab, lapatemporomandibular joint endoscopy, surgical rostomy, rhynoplasty, uncomplicated, foreign body extraction from nose - surgery, open biopsy for soft tissue tumor inside pelvis, external rhino-surgery, etc.

**Minor operations **were long leg plaster, cervical polyp excision, gastroscopic polypectomy, cervical biopsy, circumcision, colposcopy, pericariodsynthesis, etc[10-15].

### Analysis

The regional comparisons were based on the calculation of ratios for Region-1 to Region-6. Chi square linear trend analysis was used to evaluate the changes in public and private shares throughout the years. Statistical significance is achieved when the *p *value is less than 0.001.

## Results

### Hospital Capacity

The public sector was the dominant health service provider in curative care between 2001 and 2006. In 2001, the public sector accounted for approximately 92% of total bed capacity in Turkey whereas the ownership of 22.1% of all hospitals and %8.9 of all hospital beds were private during the same year.

However, the share of private sector increased to 29.4% for hospitals and 11.2% for hospital beds in 2006. There were significant changes between public and private share in number of hospitals (χ^2^_t _= 21.085, p = 0,0000), number of hospital beds (χ^2^_t _= 533,225, p = 0,0000), and number of specialist physicians (χ^2^_t _= 841,181, p = 0,0000) in 2001-2006.

Table 1 summarizes the findings for the number of hospitals per 100 000. Even though Region 1 is the most developed and affluent among the regions, the number of public hospitals per 100 000 has been the last in the rank for all years (Table 1). The number of public hospitals per 100 000 decreased for all regions throughout the years.

**Table 1:** The number of public and private hospitals according to regions and years (per 100 000)

		2001	2002	2003	2004	2005	2006
**Region 1**	Public	0.8	0.8	0.7	0.8	0.7	0.7
	Private	0.7	0.8	0.7	0.7	0.7	0.7
**Region 2**	Public	1.5	1.4	1.4	1.4	1.2	1.2
	Private	0.4	0.4	0.4	0.4	0.5	0.5
**Region 3**	Public	2.1	1.6	1.8	1.8	1.7	1.7
	Private	0.1	0.1	0.1	0.1	0.1	0.2
**Region 4**	Public	2.5	2.2	2.2	2.3	2.1	2.1
	Private	0.1	0.1	0.1	0.1	0.2	0.2
**Region 5**	Public	1.6	1.3	1.3	1.3	1.2	1.2
	Private	0.1	0.1	0.1	0.1	0.2	0.2
**Region 6**	Public	1.5	1.2	1.2	1.4	1.3	1.3
	Private	0	0	0	0	0.1	0.1

There were very few private hospitals per 100 000 in Region 3,4,5,6 in all years. The regional inequalities have persisted throughout the years for the number of public hospitals per 100 000.

Number of hospital beds and specialist physicians has shown regional differences throughout the years. For public sector, in the most developed region, the number of hospital beds per 10 000 was 20.8, while it was 8.9 in the least developed region. It was 3.8 and 0.1 for private sector, respectively. The number of public hospital beds per 10 000 remained 20.8 in region 1 for 2001 and 2006 and for the other regions the increase throughout the years was between 1.2-1.3 fold. However the increase in the number of private hospital beds has been 1.4 fold for R1, 2.0 for R2 and 3.0 for R6 between 2001 and 2006.

**Figure 2:**
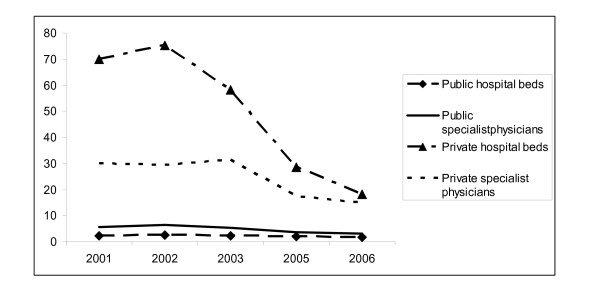
**Rate ratios of hospital beds and specialist physicians of public and private hospitals**. *At 2004 there were no data for private sector in Region 6

Figure 2 presents the rate ratios of hospital beds and specialist physicians of public and private hospitals. The rate ratio (R1/R6) of public hospital beds in R1 to R6 has changed from 2.3 to 1.8 between 2001 and 2006, but for private hospital beds this ratio has decreased from 29.8 to 14.7 as a consequence of substantial increase in private hospital beds. Regarding to the human resources capacity of the private sector the rate ratio of the number of specialist physicians decreased between 2001-2006.

### Hospital services

The utilization of all hospital services in both sectors increased between 2001-2006. The public sector had a dominant role for hospital care/services.

However, the share of public sector in the outpatient services provided between 2001 and 2006 declined from 96.4 % to 90.7% As for major, intermediate and minor surgical operations and the deliveries, the decline in the public sector service provision have been respectively as follows: from 86.6% to 79.3%. from 90.5% to 79.3%, from 88.6% to 86.1%, from 89.8% to 80.8%, between 2001 and 2006.

**Figure 3:**
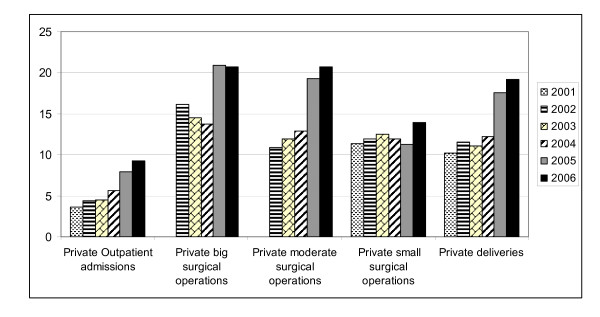
**The percentage of private services among all services in Turkey (2001-2006)**.

Whereas for the same services mentioned above the private sector' s share went up from 3.6-13.4% in 2001 to 9.3-20.7% in 2006. Figure 3 summarises these changes.

There were significant changes between public and private share in outpatient admissions (χ^2^_t _= 5709294, p = 0,0000) major surgical operations (χ^2^_t _= 34064,967, p = 0,0000), intermediate surgical operations (χ^2^_t _= 60383,140, p = 0,0000), minor surgical operations (χ^2^_t _= 902,184, p = 0,0000) and deliveries (χ^2^_t _= 36179,124, p = 0,0000) in 2001-2006.

Rate ratios (Region1/Region6) of deliveries, surgical operations per 10 000 according to sectors and years are presented in Table 2. Regarding the regional inequalities in the utilization of hospital services in private sector, the rate ratios for major, intermediate, minor surgical operations and deliveries have increased between 2001 and 2002. There was a sharp decrease in 2005 and 2006.

**Table 2:** Rate ratios (Region1/Region6) of deliveries, surgical operations per 10 000 according to sectors and years

		2001	2002	2003	2004	2005	2006
Major surgical operations	Public	6.1	6.8	4.9	4.3	4.4	3.6
	Private	117.7	209	147.1	NA	26.3	13.9
Intermediate surgical operations	Public	2.7	2.7	2.6	2.3	2.6	2.3
	Private	14.6	17	19	NA	22.9	10.2
Minor surgical operations	Public	5.5	6.8	7.5	4.3	4.1	4
	Private	46.2	73	34.8	NA	62.6	35.7
Deliveries	Public	1.1	1.1	1	0.9	0.9	0.9
	Private	27.8	45.6	39.6	NA	14.1	13.4

NA:There has been no information for private hospitals in Region 6.

In 2001 in R1 major surgical operations were performed at the public sector six times more than R6. For the same period at private sector for the same operations, the ratio between R1 and R2 was 117.7 fold. In 2006 a the ratio between the same regions for the same type of operations decreased to 3.6 for public and 13.9 for private sector.

## Discussion

This study is unique as it is the first to assess the regional differences in capacity and utilization of health services in private and public sectors in Turkey throughout 2001-2006 by analysing the only available national data for regional comparison [4].

For the implementation of effective health reforms, different forms and levels of privatization has been an important component of the solutions proposed to improve the health systems around the world. However especially in the developing world, there is limited data available to evaluate the effects and the consequences of privatization. Results of this study may help to highlight these effects of the privatization process in a developing country such as Turkey.

As shown in this study, the private sector grew so rapidly in Turkey between 2001-2006 especially after 2004 in terms of capacity and service delivery. There were substantial regional inequalities both in public and especially private sector. But after 2005, the inequalities between R1 and R6 decreased for private sector significantly while a little change was observed for public sector.

### Hospital Capacity

Turkey has fewer hospital beds per capita relative to other comparable income countries in OECD[3,18]. Regional and urban-rural inequalities exist in the access and utilisation of health services. In spite of the poor health status of rural areas and eastern part of the country, people in these areas had the most difficulty in access and received more expensive services [3]. In line with these informations, this study demonstrated that within public sector, there were 2.3 times more beds in R1 compared to R6 in 2001. The difference was 69.9 times more for the same regions at the private sector. In 2006 number of private hospitals increased substantially even in R6.

The change in capacity of public hospitals has not been parallel to the changes in private sector. Throughout these years, in all regions, number of public hospitals per capita decreased while a small increase was observed in number of public hospital beds. It can be concluded that in dealing with inequalities in access to health services not much has been achieved in the public sector during this period.

This was surprising, because within the same period, public spending on health care grew much more quickly than total spending on health care prior to HTP. Moreover the total health expenditure for curative and rehabilitative in-patient care has constituted 41% of the health budget[3,19]. By 2006, the public share of total health spending had reached to 72%, just below the OECD average of 73.3%[3]. Between 2001-2006, the share of public spending in total health spending increased from 68.2% in 2001 to 72.4% in 2006. The amount of money spent on social security increased from 34.5% to 41.0%. The share of the budget for investments improved from 3.9 % to 4.9%, while the share of private sector's spending decreased from 31.8% to 27.6% [8].

These figures show that the investments in public sector has had very little role in the increase of the overall health budget. However the increase in public share of total expenditure on health has been reflected in the increase in private sector expansion in the number of beds and number of institutions. Similar sort of experiences have taken place at earlier stages of health reform for most countries going through a transformation in their health system. In Bangladesh, in an acute crisis in access to health care, privatization has been considered as a major solution of the issue. However health sector spending continued to grow even after 1980 when generally the fiscal deficit in the state budget was growing and government was looking for ways to control expenditure. However growth in the number of beds and institutions in the public sector had slowed down by the mid-1980s. From 1986-1996, growth in the private sector surpassed that in the public sector by a wide margin[20,21].

### Hospital services

In Turkey, the economical growth in the health sector has not been mirrored in the share of the public sector. Although conserving its dominant role, the percentage of public health services has declined for outpatient admissions, major, intermediate and minor surgical operations and deliveries between the years 2001-2006. However the share of private sector within the health service has shown a considerable expansion after 2004. The ongoing health reform and some legislative changes adopted throughout these years have played an important role in encouraging the private sector to invest even at deprived regions of the country. Before July 2003, patients financed private care services by out-of-pocket payments or through their private health insurance except some cardiovascular services purchased by only SSK for its members [22]. Since then, access to private health facilities for members of public social security institutions were allowed for outpatient and inpatient services and these services were financed by the public [3,8]. Approximately 1 000 private facilities (hospitals, outpatient clinics, diagnostic centers, etc.) currently have got contracts with the SSK of which 350 are private hospitals [3] The relationship between SSK and private facilities operates under a more traditional purchaser-provider model, whereby the GHI contracts with private hospitals to deliver services included in the benefits package. Payment by SSK funds was on a retrospective basis (fee-for-service) and fee schedules and payment mechanisms across the different health providers (*i.e.* university, public and private) were not co-ordinated. Co-payments requested by private hospitals also showed diverse patterns. The coverage of the health services provided to SSK members by each of these private hospitals were different according to scope and type of the contract and hospital. This explains the rapid increase in number of surgical operations, deliveries performed in private sector.

Contracting out is another form of privatization in healthcare management and operations [1]. Outsourcing of hospital clinical services (diagnostics) to the private sector also make substantial contributions to the growth of the private sector[5]. In a study conducted in five big cities in Turkey including 80 hospitals, the following services were found to be outsourced: hospital management information systems (83.8%), cleaning services (81.3%), maintenance services (72.5%), leased medical devices (75.0%), food services (60.0%), patient direction services (63.8%), magnetic imaging services (60.0%), other imaging services (48.8%), laboratory services (42.5%)[23].

Provider payment methods, such as allowing private hospitals to implement "extra billing" were adopted by the SSK to stimulate private sector interest in contracting with the SSK. In 2006, a Public-Private Partnership (PPP) Law for the health sector was adopted and a new PPP unit was set-up under the MoH, mandated to pilot PPPs in the health sector [3].

### Inequalities

The regional inequalities for the inpatient services have changed for public sector as well as private sector. However, the level of change has been remarkably different. While private sector had an enormous growth even in less developed regions, the rate ratios were almost stable in public sector over the years, despite the fact that the public sector has a dominant role in health service delivery. This was an unexpected finding because one of the key elements of HTP include expanding the delivery of health care and to improve equity in access to health services [3].

Private sector expanded with a substantial resource allocation from public especially from social security institutions [3]. Private health services are only available to those who have got the financial means to pay for them, who have private insurance or some form of public insurance [3]. However according to Turkey National Health Accounts Household Health Expenditures 2002-2003 report, the percentage of uninsured were 35.5%, and the limited social security programme established for poor (Green Card holders) covered 10.1%. In least developed regions, the percentage increased to 47.5 for the uninsured [24]. These groups have to spend out of pocket payments for private health services. The effect of rapid expansion of the private sector on inequalities has been an important concern in reports about Turkey [24]. There have been studies suggesting that health care accessibility became less equal as an effect of privatization [25-27]. Thus how the enlargement in private sector will contribute to reducing the inequalities in access to health care is questionable.

#### Study limitations

The possibility of non registered private hospitals, informal service use, under notification of service use to the MoH may bias the share of private sector.

This is a retrospective study based on records, it has similar characteristics of ecologic studies and analytic structure is limited.

Another limitation of this study is the use of rate ratios for measuring inequalities. From the point of view of monitoring health inequalities and evaluating policy interventions, it is very important to estimate relative and absolute differences[28]. When assessing inequalities, the available measures differ substantially in degree of sophistication. Simple measures such as rate ratios or rate differences between a lower and a higher group have the advantages of easy calculation and straightforward interpretation and do not pose many restrictions on the data used in calculation. Such measures of effect as the rate ratio (a relative measure) appear to demonstrate the increase or decrease in inequalities[29]. However, they ignore some parts of the available information. The rate ratio measures- while comparing the highest versus lowest groups- do not take into account the rates *in-between *the highest and lowest groups. This limitation of simple measure of inequalities is also present in our study [29].

## Conclusions

The private sector, motivated by the generous purchase policies which transferred public budget to private health services has grown enormously between 2001-2006- especially after 2004-even at the less developed regions of Turkey. Although this serves as a mediating factor for diminishing inequalities in the use of private health services it is important to take into consideration that private sector does not have a leading role in service delivery. The fact that, a lot of uninsured people lives in these underdeveloped regions, should not be avoided. When these are taken into account, the enlargement in private sector is not an insightful solution that may contribute to diminishing the inequalities in access to health care. The inequalities have persisted throughout the years for public facilities in number and in capacity inspite of its fundamental role in service delivery and this is contradictory. In fact, this enormous growth in private sector should be seen- especially among low income groups of low developed regions- a relevant booster of the already high health inequalities.

## Competing interests

The authors declare that they have no competing interests.

## Authors' contributions

HADA participated in the conception and design of the study; analysis and interpretation of the data; and drafting the paper or revising it critically for substantial intellectual content to the manuscript, IE participated in the conception and design of the study; analysis and interpretation of the data; and drafting the paper or revising it critically for substantial intellectual content to the manuscript ZO participated in the conception and design of the study; interpretation and drafting the paper or revising it critically for substantial intellectual content to the manuscript. All authors read and approved the final manuscript.

## Pre-publication history

The pre-publication history for this paper can be accessed here:

http://www.biomedcentral.com/1472-6963/10/300/prepub
